# Transconjunctival Orbitotomy as Surgical Management of an Orbital Lipoma in a Dog

**DOI:** 10.1111/vop.70261

**Published:** 2026-08-27

**Authors:** Lucas de Souza Viana, Francis Arthur Seco Prando, Isabela Pessôa Barbieri Bastos, Adriano de Sales Araujo, Bruno Alberigi

**Affiliations:** ^1^ Programa de Residência Em Medicina Veterinária, Instituto de Veterinária (IV) Universidade Federal Rural Do Rio de Janeiro (UFRRJ) Rio de Janeiro Brazil; ^2^ Programa de Pós‐Graduação Em Medicina Veterinária, Instituto de Veterinária (IV) Universidade Federal Rural Do Rio de Janeiro (UFRRJ) Rio de Janeiro Brazil; ^3^ Departamento de Medicina e Cirurgia Veterinária (DMCV), Instituto de Veterinária (IV) Universidade Federal Rural Do Rio de Janeiro (UFRRJ). Rio de Janeiro Brazil

**Keywords:** atypical lipoma, canine, ocular oncology, orbital disease, veterinary ophthalmology, veterinary surgery

## Abstract

Orbital lipomas are rare in dogs and may pose diagnostic and surgical challenges. This report describes the case of a 14‐year‐old female Border Collie presenting chronic exophthalmos caused by a retrobulbar mass. On ophthalmic examination, the animal maintained bilateral vision despite chronic exposure of the right ocular surface. Computed tomography revealed a retrobulbar mass with density compatible with adipose tissue. Cytological examination was performed as a screening test and supported the diagnostic suspicion. An anterior subconjunctival orbitotomy was performed, ensuring preservation of the dog's eye and vision. Cytology and histopathological examination, correlated with the clinical history, confirmed the diagnosis of an orbital lipoma. This case highlights the importance of advanced imaging for surgical planning and supports the use of minimally invasive orbitotomy techniques for selected benign orbital lesions, prioritizing functional and aesthetic preservation of the eye.

## 1 Introduction

The ocular orbit is composed of the bones that surround the eye, orbital fat, fascial planes, extraocular muscles, and afferent and efferent nerves that supply the orbital contents or simply pass through the orbit to innervate the nose or other fascial structures [1]. In dogs and cats, the orbit is open, characterized by an orbital margin composed of bony components and the orbital ligament [2]. Orbital diseases commonly produce a painful condition when opening the mouth, exophthalmos, protrusion of the nictitating membrane, periocular inflammation, and deviation of the globe, although these overlapping clinical signs often make the underlying etiology difficult to determine on examination alone [2, 3]. An accurate diagnosis is essential to establish the correct management and prognosis of the case [3].

The causes of exophthalmos are most often inflammatory or neoplastic, although other conditions (e.g., cystic, vascular, or traumatic) may also occur. Ocular neoplasms are relatively uncommon in dogs and may have a significant impact on vision, comfort, and longevity [4]. In the orbit, malignant tumors are the most frequent, whereas benign neoplasms represent a minority [5].

Lipomas are benign neoplastic masses composed of adipocytes [6]. The involvement of this neoplasm in ocular tissues is atypical. Orbital lipoma is a rare condition with a strong potential to generate relevant clinical signs of retrobulbar disease [7]. Although cutaneous and subcutaneous lipomas have a common clinical occurrence [6], there are reports of this mesenchymal tumor in other atypical locations besides the orbit, such as the lung lobe and the abdominal cavity [8, 9]. Overweight, elderly, and neutered dogs tend to have a greater likelihood of developing the disease [6].

The diagnostic approach and surgical management of orbital lipomas remain poorly described in veterinary medicine, particularly regarding techniques that allow preservation of the globe and visual function [7, 10]. This study aims to contribute to the understanding of orbital diseases in dogs by reporting the first case of orbital lipoma in Brazil.

## 2 Case Report

A 14‐year‐old female Border Collie weighing 15.9 kg, presenting with a mass in the right eye, was examined at the ophthalmology service of the veterinary hospital at the Federal Rural University of Rio de Janeiro. The dog's caregiver sought a second veterinary opinion, reporting a swollen appearance in the right eye over a 10‐month period, with rapid progression in the last 6 months. The dog did not show significant discomfort, changes in feeding habits, or systemic diseases. A computed tomography scan of the skull had been previously performed, and the dog had been receiving dorzolamide hydrochloride 2% ophthalmic solution (EMS, São Paulo, Brazil; once daily in the right eye) and prednisolone 5 mg (oral administration, approximately 0.3 mg/kg, once daily) for 10 days.

**FIGURE 1:**
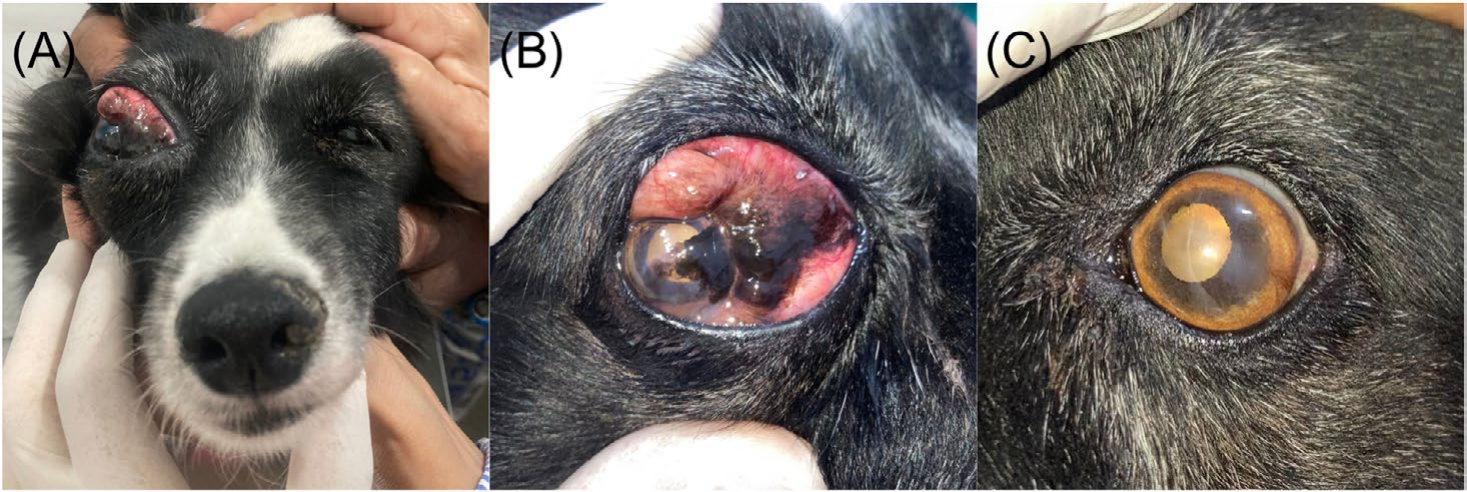
Dog with evident exophthalmos in the right eye (A). The affected right eye is shown in greater detail (B), and the intact left eye (C).

Ophthalmic examination revealed exophthalmos and lagophthalmos in the right eye (Figure 1). The menace and dazzle reflex tests were positive in both eyes. Retropulsion decreased in the right eye (OD) compared with the contralateral eye. Slit‐lamp biomicroscopy (Portable Slit Lamp Apramed) of the right eye revealed a hyperemic bulbar conjunctiva with a marked, diffuse swelling, soft consistency, pink coloration with darkened pigments, and partially reducible upon palpation. The cornea showed corneal pigmentation due to exposure keratitis in the nasal quadrant and corneal opacities. In the left eye (OS), only mild Florida spots were observed, characterized by round, grayish‐white opacities of the anterior corneal stroma consistent with Florida spot keratopathy [11]. Rebound tonometry (Tonovet tonometer) was performed, with values of 20 mmHg in OD and 15 mmHg in OS. Fundoscopic examination performed with a binocular indirect ophthalmoscope and a Volk 20‐diopter lens revealed an intact posterior segment in both eyes.

**FIGURE 2:**
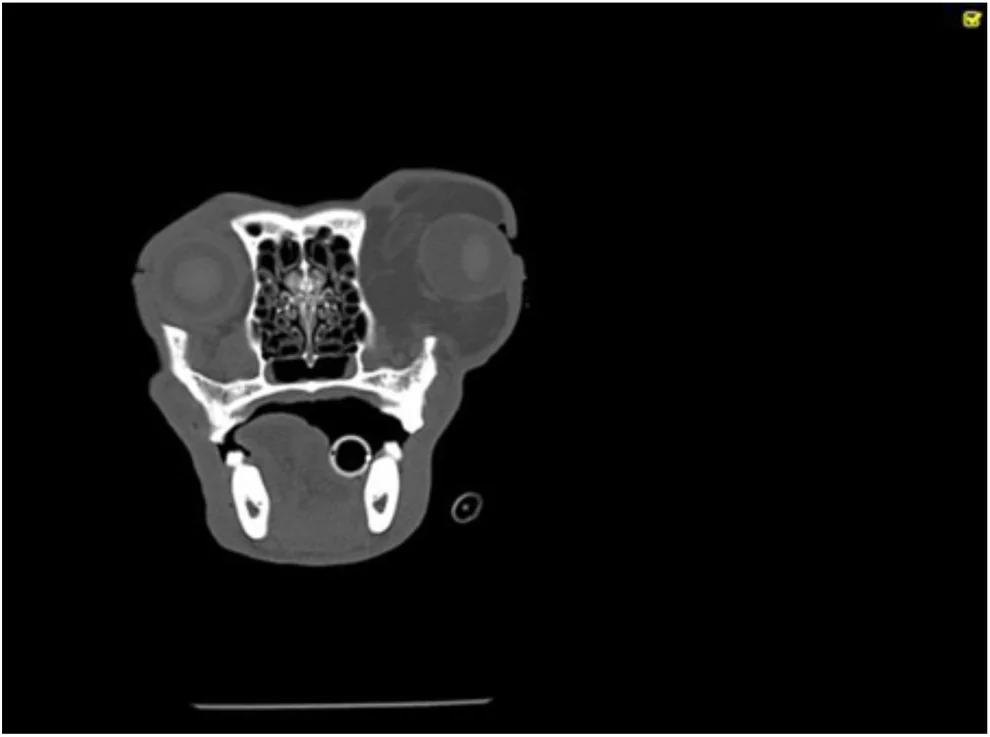
Computed tomography of the dog's skull.

Cytological samples of the bulbar conjunctival mass were obtained through fine‐needle aspiration (FNA), performed without ultrasound guidance, characterized by a normocellular sample composed of three‐dimensional clusters of mature adipocytes. Computed tomography (CT) of the skull (Figure 2), which had been performed previously, revealed a well‐defined mass measuring 4.4 cm in height and 3.5 cm in width, with fat density suggestive of a lipoma in the peribulbar and retrobulbar region of the right eye. This mass did not invade ocular and/or intracranial structures. Based on these findings, an anterior subconjunctival orbitotomy was indicated for the dog, followed by histopathological submission of the excised mass. Thoracic radiography, electrocardiography, echocardiography, and hematological and biochemical tests were requested as preoperative examinations. No noteworthy alterations were observed in the preoperative tests, and the dog was cleared for the surgical procedure. Blood analysis revealed mild hyperproteinemia (total protein 8.2 g/dL), lipemic plasma (+), and elevated albumin (3.6 g/dL).

**FIGURE 3:**
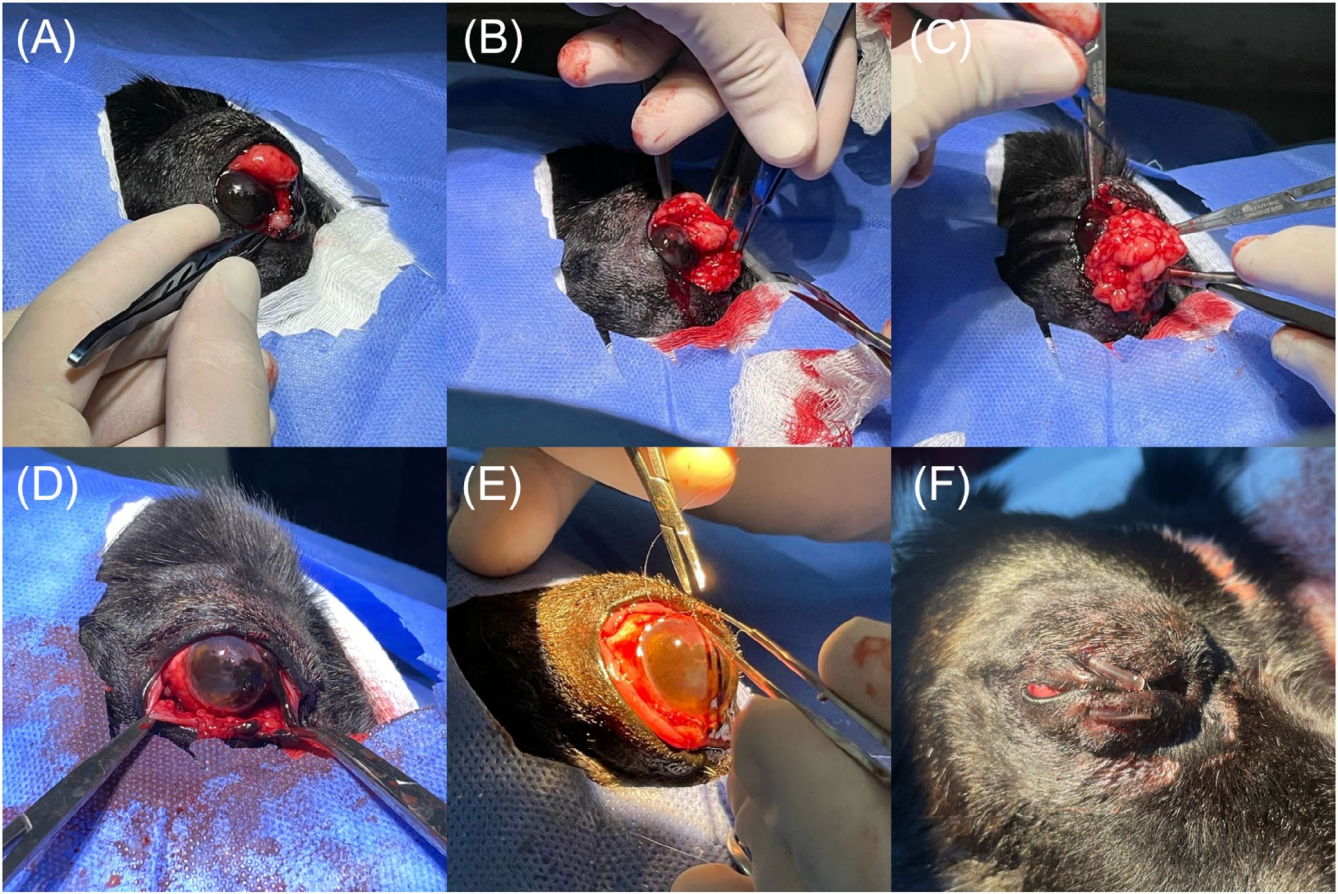
Anterior subconjunctival orbitotomy. Subconjunctival blunt dissection of the mass through a perilimbal approach in the bulbar conjunctiva (A, B, and C), followed by excision of the mass (D), bulbar conjunctival suturing (E), and temporary partial tarsorrhaphy (F).

The dog underwent surgical excision of the mass in the peribulbar and retrobulbar region (Figure 3). The dog was maintained under general anesthesia following endotracheal intubation. The dog was positioned in ventral recumbency and the periocular region was clipped. Standard surgical antisepsis was performed using sterile gauze and washes of 0.5% povidone‐iodine solution. After preparation of the surgical field, the procedure was initiated. A perilimbal incision of approximately 12 mm was made in the dorsal conjunctiva, followed by subsequent subconjunctival blunt dissection. The dissection allowed direct access to the encapsulated peribulbar mass located between the conjunctiva and the sclera, permitting fragmentation and excision in blocks. As the peribulbar content was excised, the retrobulbar mass projected into the peribulbar region and was also excised. It was not possible to visualize and isolate the medial rectus muscle, which may have been ruptured during surgical manipulation. The nictitating membrane remained intact. After excision of the mass, the dog's exophthalmos resolved during the intraoperative period. The bulbar conjunctiva was closed using a simple interrupted pattern for anchorage, followed by a simple continuous pattern, using 8–0 polyglycolic acid suture material. The surgical procedure was concluded with the performance of a temporary partial tarsorrhaphy using 4–0 nylon suture in a horizontal mattress pattern placed over stents. All fragments of the mass were submitted for histopathological examination. The anesthetic intraoperative period remained stable, and the dog maintained heart rate and systemic arterial pressure within normal limits throughout the procedure. The dog recovered from anesthesia without complications and continued postoperative treatment at home, which consisted of rest, use of an Elizabethan collar, and topical and systemic medications.

Postoperative medications included tobramycin 0.3% ophthalmic solution (Tobrex, Alcon, São Paulo, Brazil) every 6 h for 14 days, sodium hyaluronate 0.15% ophthalmic solution (Hyabak, Laboratoires Théa, Loures, Portugal) every 12 h for continuous use, prednisolone 10 mg (Predsim, Mantecorp Farmasa, São Paulo, Brazil; 3/4 tablet, 0.5 mg/kg) every 24 h for 7 days, tranexamic acid 250 mg (Transamin, Eurofarma, São Paulo, Brazil; 1/2 tablet, 8 mg/kg) every 12 h for 3 days, amoxicillin trihydrate with potassium clavulanate 250 mg (Agemoxi CL, Agener União, São Paulo, Brazil; 1 tablet, 15 mg/kg) every 12 h for 14 days, and dipyrone 500 mg (Medley, São Paulo, Brazil; 3/4 tablet, 25 mg/kg) every 8 h for 5 days. Tranexamic acid was prescribed to prevent and control postoperative bleeding, given the vascular nature of the orbital surgical bed. The dog remained stable, and the eye appeared comfortable, without local swelling.

**FIGURE 4:**
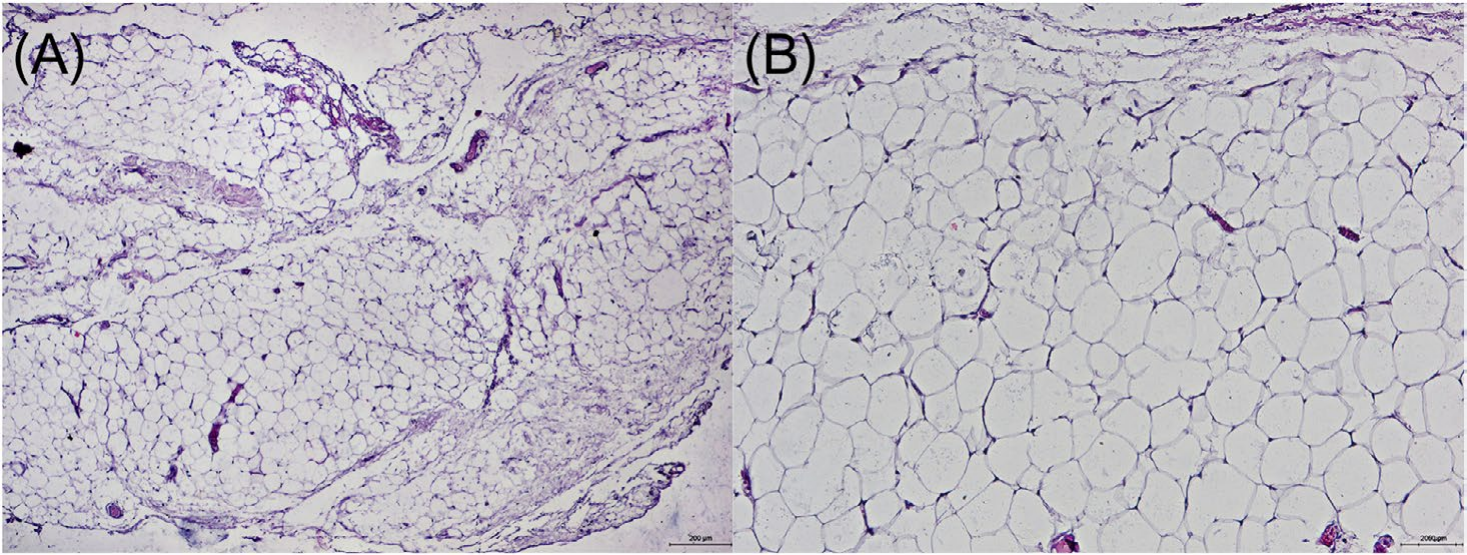
Microscopic images from histopathological examination at 10× (A) and 40× (B) magnification.

On histopathological examination, irregular, white fragments, soft to friable and cohesive, were described macroscopically. The largest measured 8.5 × 2.0 × 1.0 cm, and the smallest measured 1.5 × 0.5 × 0.1 cm. On microscopy (Figure 4), the histological sections were composed of abundant well‐differentiated adipocytes within extensive areas of hemorrhage. At the periphery of the described adipocyte population, a thin fibrous capsule was multifocally observed. Pleomorphism, anisocytosis, and anisokaryosis were mild, and no mitotic figures were observed in 10 high‐power fields (40× objective).

**FIGURE 5:**
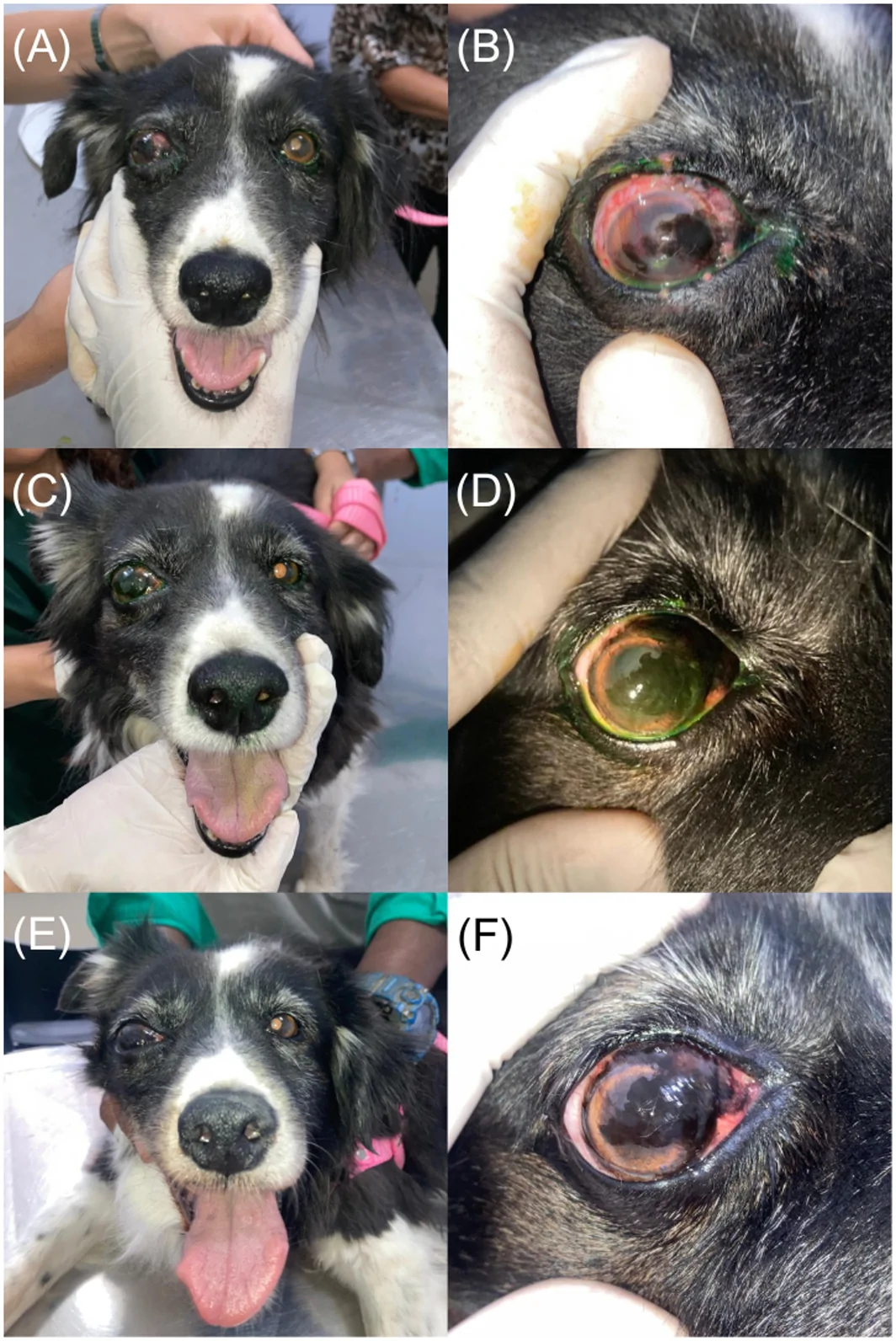
Dog during postoperative follow‐up at 21 days (A and B), 7 months (C and D), and 11 months (E and F).

The partial tarsorrhaphy was removed 21 days after the surgical procedure, and the right eye appeared visual with a marked reduction in exophthalmos (Figure 4). Two months after surgery, the dog returned for follow‐up, and a new ophthalmic examination was performed: menace response positive (both eyes), Schirmer tear test 20 mm/min (OD) and 18 mm/min (OS), intraocular pressure (IOP) 17 mmHg (OD) and 12 mmHg (OS), lateral strabismus in the right eye, partial corneal pigmentation in the right eye, and Florida spots in the left eye. The specific cause of the post‐operative strabismus was not identified by additional testing; however, the strabismus was attributed to intraoperative disruption of the medial rectus muscle as described above. The remaining findings (including neuro‐ophthalmic tests, intraocular inspection, and fundoscopic examination) did not show alterations. The dog continues to be monitored 11 months after surgery and remains comfortable, visual, and without recurrence of the ocular condition observed during physical examination (Figure 5).

## 3 Discussion

The observation of an orbital lipoma in a 14‐year‐old female dog is consistent with the findings of other authors, who report a higher occurrence of this type of neoplasm in elderly dogs [6, 12, 13]. Although exophthalmos and strabismus are nonspecific findings commonly reported in orbital diseases [3], the soft and partially reducible consistency of the mass observed in this case may represent a clinically relevant feature suggestive of a lipomatous lesion, which is not frequently emphasized in previous reports. The chronic and nonpainful progression of the condition further supported a neoplastic etiology, as inflammatory orbital diseases are typically associated with pain and acute onset [3, 7, 13, 14].

Cytological evaluation was performed as a minimally invasive and accessible diagnostic method, providing important preliminary information for clinical decision‐making and surgical planning. The identification of mature adipocytes supported the presumptive diagnosis of lipoma and was consistent with previous descriptions [12], reinforcing the role of cytology as a diagnostic screening method and for surgical planning, despite its known limitations in definitively characterizing adipocytic tumors [15, 16].

Computed tomography was essential in this case, not only for lesion detection but also for characterization and surgical planning. Coall et al. [15], in a study of 97 animals with orbital diseases, emphasized that advanced imaging examinations (CT or magnetic resonance imaging) can detect abnormalities that support the diagnosis of inflammatory or neoplastic disease and can also help identify foreign bodies. The identification of a well‐defined mass with fat attenuation strongly supported the presumptive diagnosis of lipoma and directly influenced the decision to pursue a conservative surgical approach with globe preservation. These findings are consistent with previously described tomographic characteristics of adipose tumors [17], highlighting the value of CT as a decision‐making tool in orbital diseases.

Prolapse of orbital fat should be considered as a differential diagnosis when examining an animal presenting clinical signs of increased volume in the bulbar conjunctiva with a fatty appearance, similar to that reported [10, 18]. In the present report, the dog showed progressive enlargement of the orbital lesion, a marked mass effect, and exophthalmos, findings consistent with the lipoma cases described by Williams and Haggett [10] and Charnock et al. [7].

The surgical management of orbital neoplasms primarily involves the decision to preserve the ocular globe or perform exenteration. In this case, the presumed benign nature of the lesion, supported by imaging and cytology, justified a conservative approach aimed at preserving vision and ocular structures. Surgical excision of the mass should be performed whenever the anatomical location permits, and different surgical approaches to orbitotomy are possible depending on the position, extent, and consistency of the lesion [12]. When available, three‐dimensional (3D) printing may be a valuable tool to improve surgical planning in cases of orbital and periorbital masses [13].

Among the orbitotomy approaches, an anterior subconjunctival approach was chosen because it is a less invasive surgical technique and still allows adequate access given the fluidity of the fatty mass, resulting in a satisfactory outcome similar to that obtained by Williams and Haggett [10]. Our experience with anterior orbitotomy for the management of lipoma corroborates the findings of Caro‐Suarez et al. [12] for this technique, as it allows adequate tumor exposure without extensive disruption of orbital tissues, ensures reversal of exophthalmos, and avoids large osteotomies. This approach provided effective tumor removal and immediate resolution of exophthalmos, corroborating previous reports that describe its utility in selected orbital lipomas [10, 12]. However, this technique requires careful case selection, as intraoperative manipulation of orbital structures may pose a risk to vision [7, 12].

Other surgical options include lateral and transfrontal orbitotomies combined with osteotomies. However, these procedures are generally more invasive, require longer surgical times, and often necessitate axial pattern flaps to reconstruct large cutaneous defects [19]. The choice of surgical approach should be tailored to the anatomical location of the orbital lipoma [12].

Histopathological examination remains the gold standard for confirming orbital neoplasms [16] and should be correlated with the dog's clinical history, clinical findings, and imaging results. In our report, the histopathological architecture of well‐differentiated adipocytes and a thin fibrous capsule is consistent with that described by Dorbandt et al. [13] and Williams and Haggett [10], both reported as orbital lipoma.

## 4 Conclusion

This report demonstrates that the anterior subconjunctival orbitotomy technique was successfully used to remove the lesion in this dog, allowing preservation of vision and satisfactory long‐term outcomes. Early integration of advanced imaging and cytology is essential for appropriate case selection and surgical planning.

## Author Contributions

**Isabela Pessôa Barbieri Bastos:** investigation, writing – original draft, writing – review and editing. **Adriano de Sales Araujo:** investigation, writing – original draft, methodology, writing – review and editing, formal analysis, data curation. **Lucas de Souza Viana:** conceptualization, investigation, writing – original draft, methodology, writing – review and editing, formal analysis, data curation. **Bruno Alberigi:** conceptualization, investigation, writing – original draft, methodology, validation, writing – review and editing, formal analysis, data curation, supervision. **Francis Arthur Seco Prando:** investigation, writing – review and editing, writing – original draft.

## Funding

This work was supported by Coordenação de Aperfeiçoamento de Pessoal de Nível Superior, Code 001.

## Ethics Statement

This case report describes the clinical management of a client‐owned dog; formal ethics committee approval was therefore not required. All diagnostic and surgical procedures were performed with the owner's authorization, and written informed consent was obtained from the owner for the publication of the animal's clinical data and images.

## Conflicts of Interest

The authors declare no conflicts of interest.

## Data Availability

The data that support the findings of this study are available from the corresponding author upon reasonable request.
